# Retos de la COVID-19

**DOI:** 10.1515/almed-2020-0100

**Published:** 2020-11-11

**Authors:** Ignacio López-Goñi

**Affiliations:** Departamento de Microbiología y Parasitología, Universidad de Navarra, 31008, Pamplona, España

El 30 de enero de 2020 aparecía en *Nature* un comentario sobre el número de artículos que ya se habían publicado acerca del nuevo coronavirus chino: más de cincuenta en menos de un mes, toda una hazaña [1]. A mediados de octubre, ya eran más de 60 mil los artículos sobre el virus SARS-CoV-2 o la enfermedad COVID-19 en PubMed. Se ha publicado más sobre el nuevo coronavirus en nueve meses que sobre otras enfermedades en lustros. Tal cantidad de información científica es imposible de analizar y estudiar con detenimiento. Entre todos esos *papers* hay auténticas basuras, que favorecen interpretaciones erróneas e incluso bulos mal intencionados. Pero también ahí puede estar la solución a muchas de nuestras preguntas e incertidumbres. Rebuscando entre la bibliografía, destaco algunos artículos, no son los mejores, pero al menos aportan algo de luz o sugieren nuevas vías para luchar contra la pandemia en aspectos tan importantes como la transmisión del virus, el diagnóstico, la inmunidad o la coincidencia del SARS-CoV-2 con otros patógenos respiratorios.

El papel que juegan el contacto a través de fómites, las pequeñas gotículas o los aerosoles en la transmisión del SARS-CoV-2 sigue siendo motivo de debate. Parece ser que el SARS-CoV-2 no se transmite con la facilidad del virus del sarampión, el campeón de la transmisibilidad aérea. Pero no cabe duda de que nos enfrentamos a un virus de transmisión respiratoria. La diferenciación entre transmisión por gotículas o por aerosoles no deja de ser artificial, una clasificación humana. Muy probablemente haya toda una graduación desde las gotículas más grandes a los aerosoles más diminutos, y las propiedades físico-químicas de la partícula viral condicionen cómo se transmite. Los virus en gotículas (mayores de 100 micras) normalmente se depositan en las superficies en pocos segundos a una distancia menor de unos dos metros. La distancia física y la higiene reducen la exposición a estas gotículas. Los virus en aerosoles (menores de 100 micras) pueden permanecer en suspensión en el aire hasta varias horas y pueden ser inhalados. Un buen símil sería el humo del tabaco. El virus transmitido por aerosoles puede viajar distancias de más de dos metros y puede acumularse en lugares cerrados y mal ventilados, dando lugar a eventos súper propagadores. Algunos estudios que demuestran la presencia del virus en superficies mediante la detección del ARN de SARS-CoV-2 y por cultivo en células Vero E6, sugieren que la probabilidad de transmisión a través de superficies es menos frecuente de lo que hasta el momento se pensaba [2]. Por otro lado, existe una abrumadora evidencia de que la inhalación del virus SARS-CoV-2 representa la ruta de transmisión más importante de la enfermedad COVID-19 [3]. Una persona con COVID-19 puede liberar gotículas y aerosoles que contienen el virus cuando respira y habla. Por todo esto, además de las recomendaciones de las 3 M -mascarillas, metros (distancia social) y manos (higiene)-, para evitar la transmisión es fundamental promover actividades al aire libre y reducir o evitar la presencia en lugares cerrados, mal ventilados, muy concurridos, durante mucho tiempo donde la gente hable en voz alta.

Las tecnologías de diagnóstico basadas en el sistema de edición genética CRISPR están revolucionando la detección de patógenos virales y bacterianos. Un ejemplo es el método SHERLOCK (del inglés, *specific high-sensitivity enzymatic reporter unlocking*) para la detección específica de ARN/ADN a concentraciones attomolar [4]. Recientemente se ha publicado un método derivado de esta tecnología, pero mucho más rápido y sencillo, capaz de realizarse a una única temperatura, en menos de una hora, con un equipamiento mínimo, y con una sensibilidad y especificidad similar a la RT-qPCR, que denominan STOP (*SHERLOCK testing in one pot*) [5]. Esta técnica combina una sencilla extracción magnética del ARN viral con una amplificación isotérmica a 60 °C y la detección mediante CRISPR-Cas12b. El límite de detección de este método es de unas 100 copias del ARN del coronavirus por reacción. Este tipo de tecnologías pueden ser empleadas en sencillos laboratorios clínicos y pueden revolucionar el diagnóstico de SARS-CoV-2 en los próximos meses.

La inmunidad de rebaño, la fracción de la población que necesita estar inmune para prevenir la extensión de una epidemia, ha sido uno de los temas más discutidos durante la pandemia. Todavía es motivo de debate si la exposición previa a los otros coronavirus humanos que causan los resfriados comunes (HCoV-OC43, HCoV-HKU1, HCoV-229E y HCoV-NL63) puede proporcionar cierta inmunidad al SARS-CoV-2. Recientemente se ha demostrado la presencia de células T memoria CD4+ que dan reacción cruzada con SARS-CoV-2 en individuos no expuestos a este virus. Lipsitch y col [6] han publicado un sugerente trabajo en el que proponen cuatro posibles escenarios sobre el impacto epidemiológico que podría tener la reacción cruzada entre los coronavirus tanto a nivel individual como colectivo. En su propuesta se basan en el efecto sobre la replicación del SARS-CoV-2 en las vías respiratorias altas y en pulmón, en la transmisibilidad del virus y en la severidad de la enfermedad COVID-19. En definitiva, se trata de incluir en los modelos y predicciones sobre el futuro de la pandemia, el papel que podría jugar una cierta inmunidad previa al coronavirus en la población.

Existe una seria preocupación sobre cómo se va a comportar el solapamiento de SARS-CoV-2 con otros patógenos respiratorios frecuentes en los meses de invierno. No se puede descartar una situación de “tormenta perfecta” en la que coincidan SARS-CoV-2 con otros coronavirus, los virus de la gripe, el respiratorio sincitial y otros que causan bronquiolitis y neumonías y son responsables de frecuentes hospitalizaciones y muertes en determinados sectores de la población más vulnerable. Vásquez-Hoyos y col. [7] acaban de publicar un estudio sobre los datos de admisiones a las unidades pediátricas de cuidados intensivos en cuatro países Latinoamericanos, de niños con problemas respiratorios por infecciones virales. Han comparado los datos de 2020 con los de dos años anteriores. Sus resultados demuestran que en 2020, durante la pandemia de COVID-19, ha habido entre un 78 y un 92% de reducción en las admisiones a las UCI debidas a otros virus. En el mismo sentido, también se ha documentado una menor incidencia de gripe en países como Chile, Australia o Sudáfrica durante Junio y Agosto de este año, los meses que constituyen la época de gripe en el hemisferio sur [8]. Aunque no se pueden descartar otras causas, muy probablemente las medidas de confinamiento, el uso de mascarillas, la higiene y la distancia social, así como la disminución de viajes y una mayor campaña de vacunación antigripal han contribuido a mitigar la circulación del virus de la gripe y a reducir su impacto. Aunque no sabemos cómo se va a comportar la gripe este invierno en el hemisferio norte, estos datos son motivo de esperanza y animan a continuar con las medidas de contención.

Aunque como hemos dicho la cantidad de información científica es abrumadora y muy difícil de asimilar, la ciencia vuelve a demostrar que el conocimiento y la cooperación son las herramientas para vencer esta pandemia. Por eso, sigue habiendo motivo para la esperanza.
